# Integrated bioinformatics analysis for exploring potential biomarkers related to Parkinson’s disease progression

**DOI:** 10.1186/s12920-024-01885-9

**Published:** 2024-05-17

**Authors:** Zhenchao Huang, En’peng Song, Zhijie Chen, Peng Yu, Weiwen Chen, Huiqin Lin

**Affiliations:** 1 Department of Neurosurgery, Lingnan Hospital, Branch of The Third Affiliated Hospital of Sun Yat-Sen University, No 2693, Kaichuang Avenue, Huangpu District, Guangzhou, 510530 Guangdong China; 2 Guangzhou BiDa Biological Technology CO., LTD, Guangzhou, 510530 Guangdong China

**Keywords:** Parkinson, s disease, ceRNA network, circRNA, Dopaminergic neurons, Hub genes

## Abstract

**Background:**

Parkinson’s disease (PD) is a progressive neurodegenerative disease with increasing prevalence. Effective diagnostic markers and therapeutic methods are still lacking. Exploring key molecular markers and mechanisms for PD can help with early diagnosis and treatment improvement.

**Methods:**

Three datasets GSE174052, GSE77668, and GSE168496 were obtained from the GEO database to search differentially expressed circRNA (DECs), miRNAs (DEMis), and mRNAs (DEMs). GO and KEGG enrichment analyses, and protein–protein interaction (PPI) network construction were implemented to explore possible actions of DEMs. Hub genes were selected to establish circRNA-related competing endogenous RNA (ceRNA) networks.

**Results:**

There were 1005 downregulated DECs, 21 upregulated and 21 downregulated DEMis, and 266 upregulated and 234 downregulated DEMs identified. The DEMs were significantly enriched in various PD-associated functions and pathways such as extracellular matrix organization, dopamine synthesis, PI3K-Akt, and calcium signaling pathways. Twenty-one hub genes were screened out, and a PD-related ceRNA regulatory network was constructed containing 31 circRNAs, one miRNA (miR-371a-3p), and one hub gene (*KCNJ6*).

**Conclusion:**

We identified PD-related molecular markers and ceRNA regulatory networks, providing new directions for PD diagnosis and treatment.

**Supplementary Information:**

The online version contains supplementary material available at 10.1186/s12920-024-01885-9.

## Introduction

Parkinson’s disease (PD) is a progressive neurodegenerative disease affecting more than 10 million people worldwide [1]. Its main pathological features are the degeneration of dopaminergic neurons in substantia nigra and the presence of neuronal Lewy bodies [2]. The disease is clinically manifested by motor symptoms including rest tremor, bradykinesia, muscular rigidity, and postural instability, often accompanied by sleep disturbance, cognitive impairment, and other non-motor symptoms [3]. Tremendous advances have been made in PD treatment, including medications such as levodopa and surgical interventions like deep brain stimulation, which effectively relieve symptoms and improve patients’ quality of life [4]. Nonetheless, specific disease-modifying treatment is still lacking [5]. Clinical diagnosis of PD mainly relies on cardinal motor symptoms, which hinders detection in the early stages of the disease [6]. It is reported that by the time PD is diagnosed, over 80% of nigral dopaminergic neurons have been degenerated [7]. Hence, identifying reliable predictive biomarkers for PD may help to improve timely diagnosis and treatment of the disease.

Emerging evidence has illuminated that noncoding RNAs such as circular RNAs (circRNAs) and microRNAs (miRNAs) work as pivotal regulators in various disorders, including PD [8]. Although they cannot code for proteins, they can regulate gene expression and participate in a wide range of biological processes, such as aging, inflammation, and neurodegeneration [9]. Moreover, it is well-established that circRNAs can act as competing endogenous RNAs (ceRNAs) to impact downstream mRNA stability and translation by competitively interacting with the shared miRNAs [10]. The circRNA-miRNA-mRNA ceRNA networks have been indicated to play critical roles in the pathogenesis and development of various diseases, including PD [11].

In recent years, microarray and sequencing technologies have been widely employed for disease biomarker screening and molecular mechanism research, which provide an ideal way to comprehensively screen disease-related genes and understand their regulatory mechanisms by using bioinformatics analysis [12]. Many bioinformatics studies have been reported to explore potential molecular markers associated with PD pathogenesis. For instance, Lei et al. identified 12 key genes related to necroptosis in PD, which might serve as novel diagnostic markers for this disease [13]. Liu et al. revealed that three hub genes including *SLC18A2*, *CALB*1, and *SYNGR3* were closely linked to immune infiltration in PD [14]. Despite these findings, understanding of the circRNA-miRNA-mRNA ceRNA networks involved in PD remains limited. Thus, further investigations are needed to help improve the understanding of circRNA-mediated ceRNA regulatory mechanisms in the pathogenesis of PD.

Herein, we downloaded three datasets from the Gene Expression Omnibus (GEO) database to identify differentially expressed circRNAs (DECs), miRNAs (DEMis), and mRNAs (DEMs) between PD and normal samples. We constructed PD-related circRNA-miRNA-mRNA ceRNA networks using various bioinformatics tools, aiming to better understand the molecular mechanisms of PD progression and screening out disease-related key genes.

## Materials and methods

### Data source

The microarray data of circRNA, miRNA and mRNA expression profiles were obtained from the GEO database (https://www.ncbi.nlm.nih.gov/geo/), a public data storage repository [15]. For the screening of circRNA expression profile, the following screening criteria were used: “Parkinson’ disease” and “circRNA”. As for the screening of miRNA dataset, the following criteria were used: “Parkinson’s disease”, “miRNA”, “*Homo sapiens*”, “expression profiling by array”, and “tissue”. The mRNA expression profile was screened using the following criteria: “Parkinson’s disease”, “mRNA”, “*Homo sapiens*”, “expression profiling by high throughput sequencing”, and “tissue”. Only datasets containing no less than 10 samples were included. Finally, the circRNA dataset GSE174052, miRNA dataset GSE77668, and mRNA dataset GSE168496 meeting the above conditions were selected for further analysis. The platform for GSE174052 is GPL28148, Agilent-084217 CapitalBio Technology Human CircRNA Array v2 [full-layout], containing 12 plasma samples (9 PD patients and 3 healthy controls). The platform for GSE77668 is GPL21437, NanoString nCounter human miRNA expression system, containing putamen samples of 12 PD patients and 12 normal controls [16]. The platform for GSE168496 is GPL18573 Illumina NextSeq 500, containing substantia nigra samples of 8 PD patients and 8 controls [17].

### Identification of DECs, DEMis, and DEMs

DECs, DEMis, and DEMs between PD and control samples were screened using the online software GEO2R (https://www.ncbi.nlm.nih.gov/geo/geo2r/). The screening criteria were set as *p* value < 0.05 and |log2fold change (FC)| ˃ 3 for DECs, *p* value < 0.05 and |log2FC| ˃ 0 for DEMis, and* p* value < 0.05 and |log2FC| ˃ 0.5 for DEMs. The results were shown in heat maps and volcano plots using the “pheatmap” package and “ggplot2” package in R software, respectively.

### Functional enrichment analysis of DEMs

For exploring the possible functional roles of the identified DEMs, Gene Ontology (GO) and Kyoto Encyclopedia of Gene and Genome (KEGG) analyses were implemented using the Database for Annotation, Visualization and Integrated Discovery (DAVID) (https://david.ncifcrf.gov/) [18, 19]. GO analysis focused on three domains: BP (biological process), CC (cellular component), and MF (molecular function). The top enriched GO terms and KEGG pathways were visualized using bubble plots and chord plots, respectively. *p* < 0.05 and gene counts ≥ 2 were regarded significant enrichment.

### Constructing protein–protein interaction (PPI) network and screening hub genes

The PPI networks were constructed using Search Tool for the Retrieval of Interacting Genes (STRING, version 11.5, https://cn.string-db.org/) [20]. A combined confidence score ˃ 0.4 was set as the cut-off value (calculated by STRING database), which was considered to indicate a significant interaction. Based on the node degrees calculated by the STRING database, the top 21 DEMs were selected as the hub genes.

### ceRNA network construction of hub genes

The Encyclopedia of RNA Interactomes (ENCORI) database (https://rnasysu.com/encori/) [21] was employed for predicting miRNAs that could interact with both DECs and hub genes. All parameters were set to default values. These predicted miRNAs were intersected with the identified DEMis, and the overlapping miRNAs were selected as key miRNAs. Then, based on the ceRNA theory, ceRNA regulatory networks of circRNAs, miRNAs, and mRNAs were constructed using Cytoscape software (version 3.10.0).

## Results

### Identification of DECs, DEMis, and DEMs

**Fig. 1:**
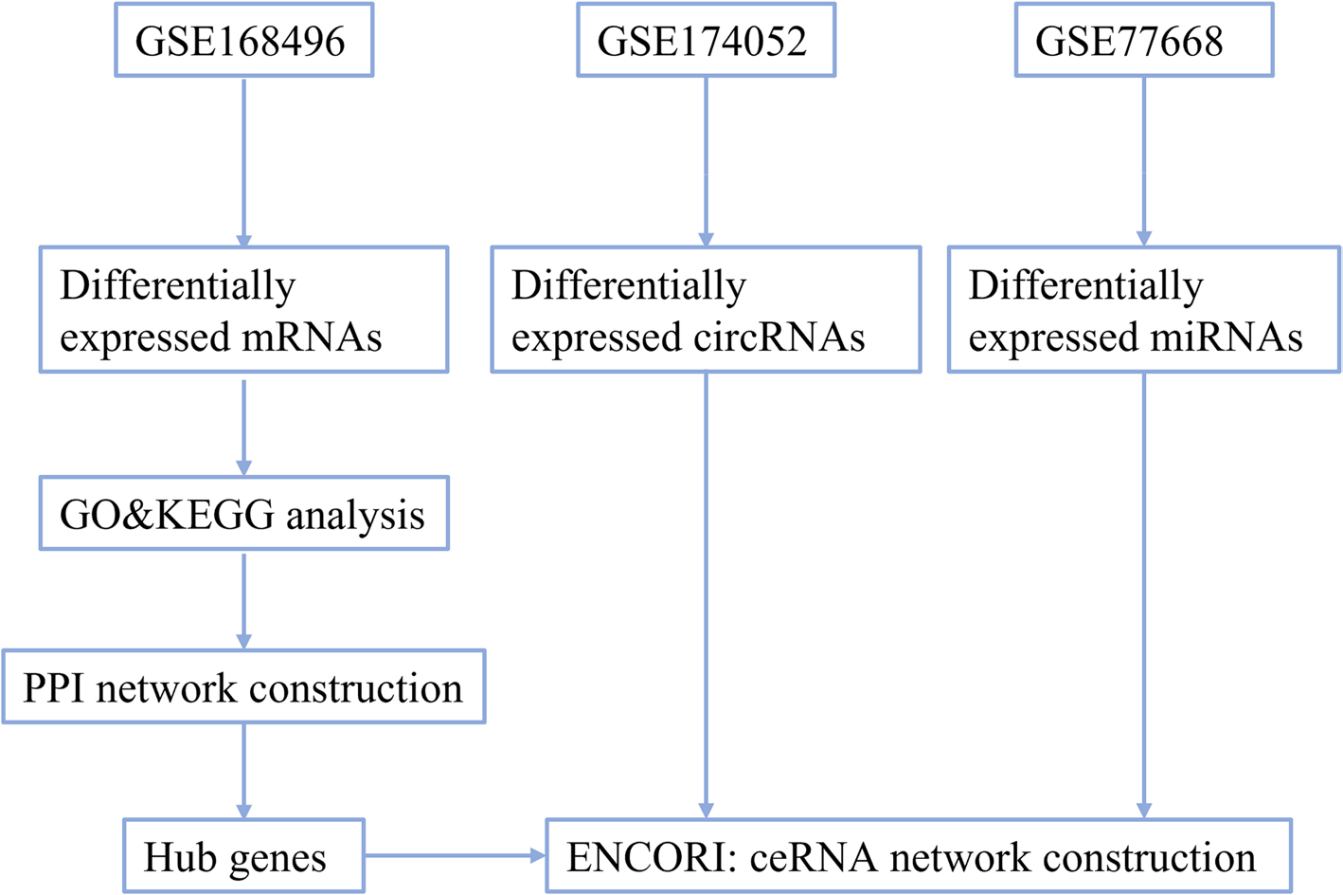
The workflow of the study

**Fig. 2:**
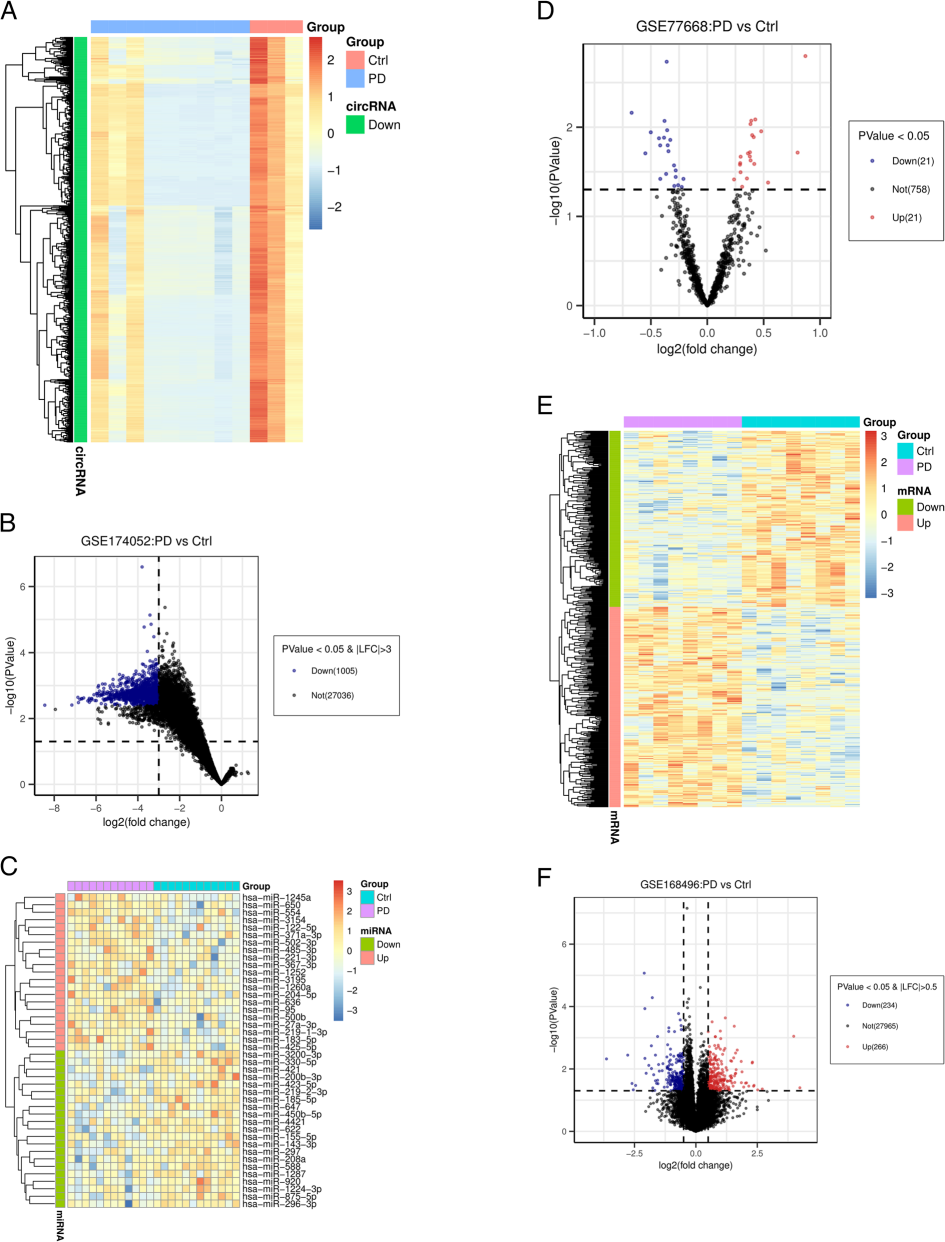
Identification of DECs, DEMis and DEMs. A-B Heatmap (**A**) and volcano plot (**B**) showing the selected DECs between PD and control samples from GSE174052 dataset. **C**-**D** Heatmap (**C**) and volcano plot (**D**) showing the selected DEMis from GSE77668 dataset. E–F Heatmap (**E**) and volcano plot (**F**) showing the selected DEMs from GSE168496 dataset

Three datasets from the GEO database were included in this study. A flowchart of the study design is shown in Fig. 1. With the screening criteria of *p*-value < 0.05 and |log2FC| ˃ 0, we identified 1005 downregulated DECs between PD and control samples from dataset GSE174052 (Fig. 2A, B). No upregulated DECs were identified from this dataset. According to the set threshold, we totally screened out 42 DEMis (21 upregulated and 21 downregulated) between PD and normal samples from dataset GSE77668 (Fig. 2C, D) and 500 DEMs (266 upregulated and 234 downregulated) from GSE168496 (Fig. 2E, F). Additionally, detailed information for the top ten dysregulated DECs, DEMis, and DEMs is shown in Supplementary Table S1-3, respectively.

### Functional enrichment analysis of DEMs

**Fig. 3:**
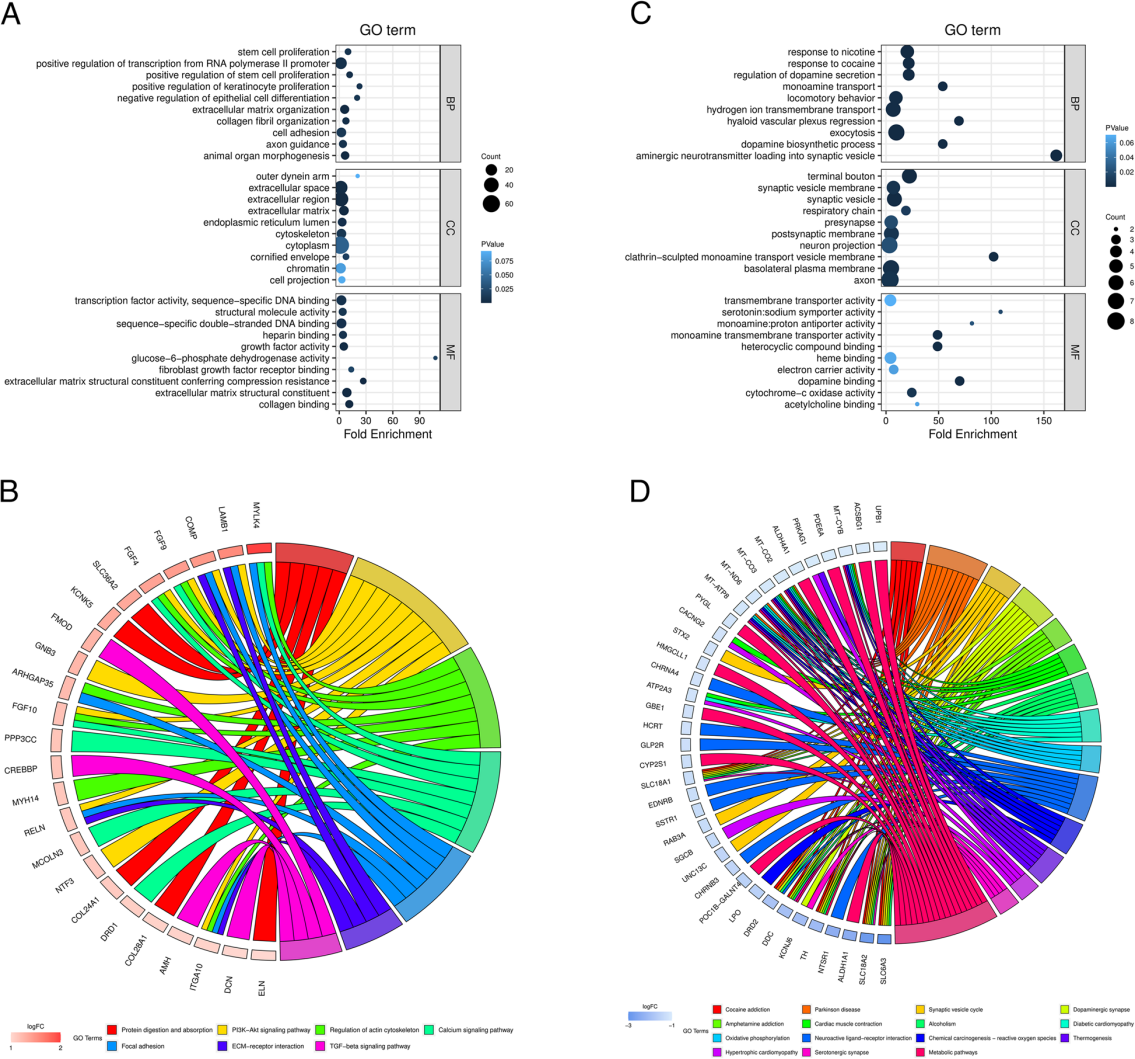
Functional enrichment analysis of DEMs. **A** GO analysis of upregulated DEMs. **B** KEGG pathway analysis of upregulated DEMs. C GO analysis of downregulated DEMs. D KEGG pathway analysis of downregulated DEMs

To explore the potential functional roles of the screened DEMs, we carried out GO and KEGG enrichment analyses. GO enrichment results revealed that the upregulated DEMs were markedly enriched in terms associated with proliferation and extracellular matrix (ECM) remodeling in the BP category, such as stem cell proliferation, ECM organization and cell adhesion; extracellular space and region in the CC category; and ECM structural constituent and collagen binding in the MF category (Fig. 3A). These results indicated that the upregulated DEMs were significantly related to ECM remodeling, which is believed to be involved in PD development [22]. Moreover, KEGG analysis also displayed the upregulated DEMs were markedly enriched in various neurodegeneration-related pathways, including PI3K-Akt signaling pathway, regulation of actin cytoskeleton, ECM-receptor interaction, calcium signaling pathway and TGF-beta signaling pathway (Fig. 3B). As for the downregulated DEMs, they were significantly enriched in response to nicotine, regulation of dopamine, dopamine biosynthetic process, aminergic neurotransmitter loading into synaptic vehicle in the BP category; synaptic vehicle membrane, axon, and clathrin-sculpted monoamine transport vesicle membrane in the CC category; and monoamine transmembrane transporter activity, heterocyclic compound binding and dopamine activity in the MF category (Fig. 3C). The above results demonstrated that the function of downregulated DEMs was closely linked to dopamine synthesis, which plays a critical role in PD pathogenesis. Consistently, KEGG analysis of the downregulated DEMs showed that they were enriched in Parkinson’s disease, dopaminergic synapse and metabolic pathways (Fig. 3D). Collectively, the above results suggested that both up- and downregulated DEMs are closely associated with PD.

### PPI network construction and hub gene screening

**Fig. 4:**
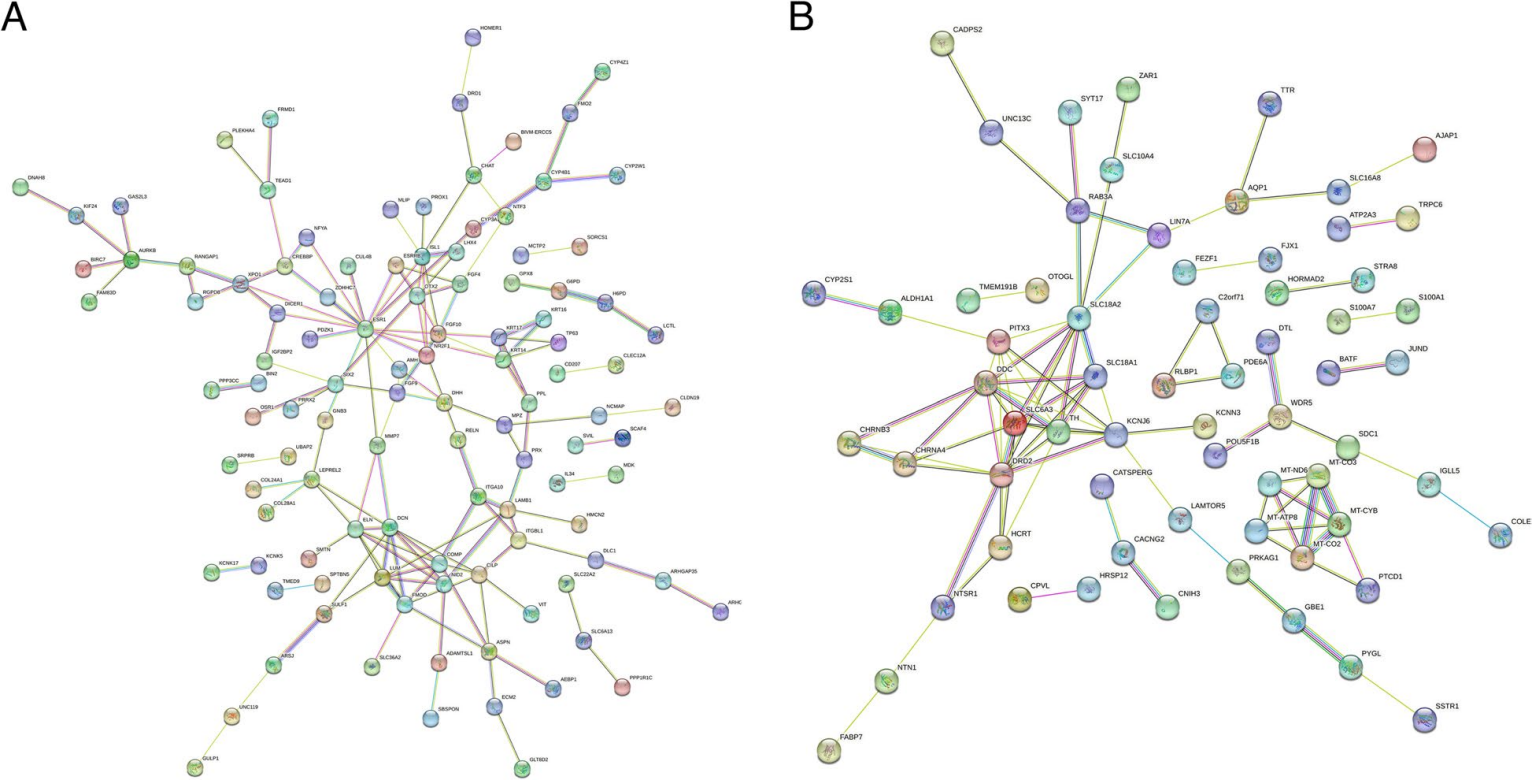
PPI network construction of DEMs. **A** PPI network for upregulated DEMs. **B** PPI network for downregulated DEMs. nodes represent genes; edges represent interaction between genes

To understand the mutual interaction of these DEMs and identify key genes involved in PD, PPI networks were constructed for the upregulated and downregulated DEMs, respectively. As displayed by the results, the PPI network for the upregulated DEMs consisted of 110 nodes and 144 edges (Fig. 4A), while that for the downregulated DEMs consisted of 66 nodes and 88 edges (Fig. 4B). Based on the node degree obtained from STRING database, we selected the top 21 genes in the two dysregulated gene groups as hub genes, including 11 upregulated genes (*ESR1*, *DCN*, *ELN*, *ISL1*, *COMP*, *FMOD*, *LUM*, *OTX2*, *CILP*, *KRT14*, *LAMB1*) and 10 downregulated genes (*DRD2*, *SLC18A2*, *DDC*, *SLC6A3*, *KCNJ6*, *TH*, *PITX3*, *MT-CO2*, *MT-CYB*, *SLC18A1*). The specific node degrees of these genes are shown in Table 1.

**Table 1:** Hub genes with high node degrees from protein–protein interaction networks

Node	Node degree	Regulation
ESR1	16	Up
DCN	9	Up
ELN	8	Up
ISL1	8	Up
COMP	7	Up
FMOD	7	Up
LUM	7	Up
OTX2	7	Up
CILP	6	Up
KRT14	6	Up
LAMB1	6	Up
DRD2	11	Down
SLC18A2	10	Down
DDC	9	Down
SLC6A3	9	Down
KCNJ6	8	Down
TH	8	Down
PITX3	7	Down
MT-CO2	5	Down
MT-CYB	5	Down
SLC18A1	5	Down

### ceRNA network construction with hub genes

**Fig. 5:**
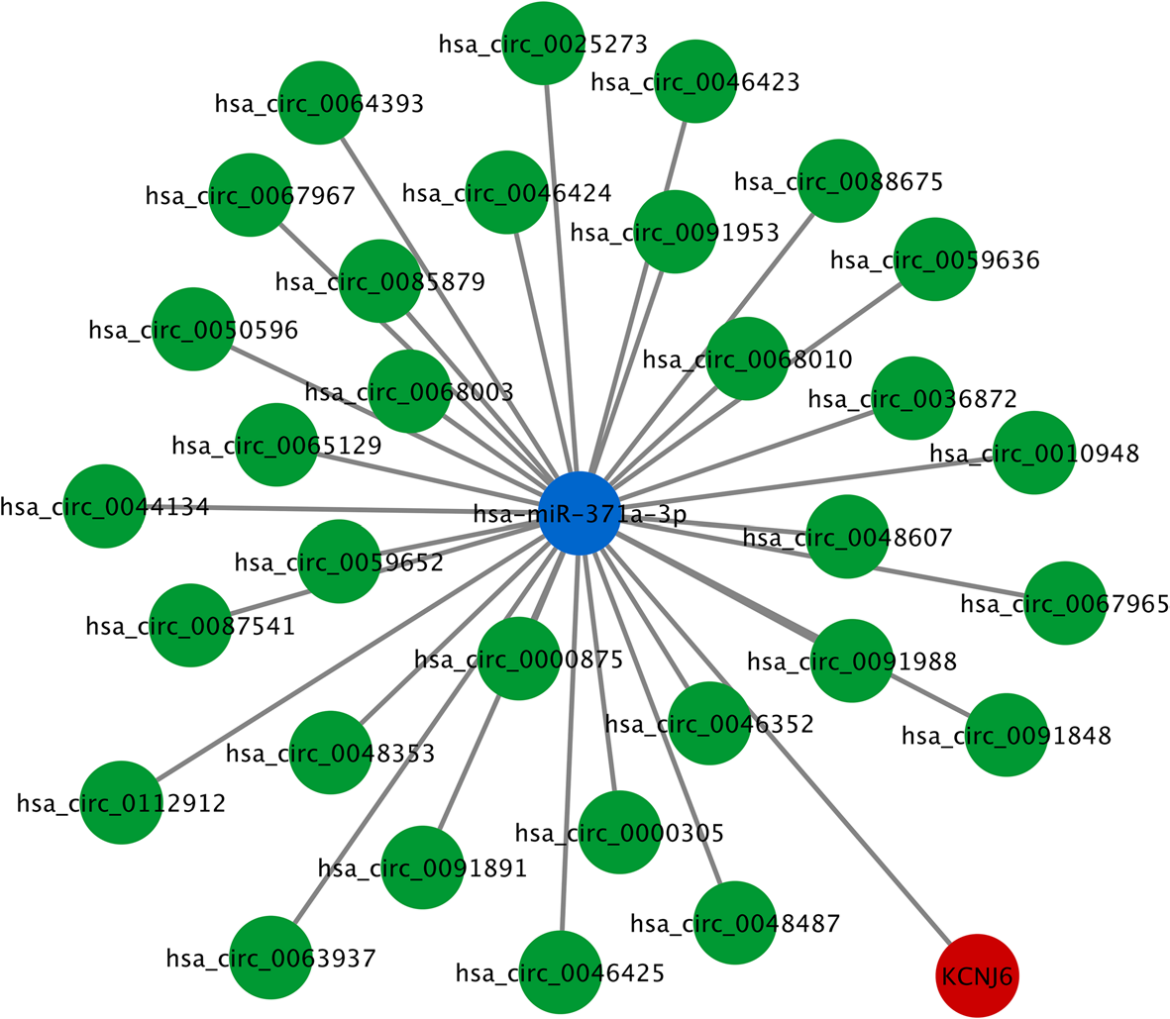
ceRNA network construction. The ceRNA regulatory network was constructed using DECs, DEMis and hub genes. Green circles represent downregulated circRNAs, the blue circle represents upregulated miRNA, and the red circle represents downregulated mRNA

To probe the potential molecular mechanism of the selected hub genes in PD, we established ceRNA networks with these genes. The ceRNA theory presents that circRNAs can upregulate mRNA expression by competitively binding to shared miRNAs [23]. Thus, considering that only downregulated DECs were identified, we predicted the upstream miRNAs of the downregulated hub genes using the ENCORI database. In addition, the downstream miRNAs of the identified DECs were also predicted. Then, the predicted miRNAs from the ENCORI database were intersected with the identified DEMis from microarray analysis, and the overlapping miRNAs in the three groups were considered to be key miRNAs. Subsequently, according to the ceRNA hypothesis, a ceRNA network of DECs, DEMis, and hub genes was constructed, which consisted of 31 circRNAs, one miRNA (miR-371a-3p), and one hub gene (*KCNJ6*) (Fig. 5).

## Discussion

PD is the second most common neurodegenerative disease, which brings great socioeconomic burdens [24]. Despite tremendous efforts, its pathogenesis remains poorly understood. Moreover, due to the lack of early diagnostic techniques, PD is usually diagnosed in later stages, leading to delays in patient treatment and poor prognosis [25]. Hence, exploring molecular pathways and novel molecular markers of PD may result in better therapy for the patients.

In this study, we explored DECs, DEMis, and DEMs from three datasets of the public GEO database. GO and KEGG analyses revealed that the screened DEMs were predominantly enriched in functions and pathways related to ECM organization, dopamine biosynthetic process, PI3K-Akt signaling pathway, calcium signaling pathway, and so on. Previous evidence has demonstrated that ECM composition is critical in shaping neuronal function and plays a pivotal role in the development of neurodegenerative diseases, including PD [26]. Moreover, studies have shown that the production of dopamine, an important monoamine neurotransmitter in the brain, exhibits both toxic and protective roles in PD [27]. The PI3K-Akt signaling pathway has been widely studied for its roles in the pathophysiological processes of the central nervous system, such as neuronal proliferation, neurogenesis, and autophagy. Plenty of evidence shows that multiple natural products protect dopaminergic neurons in PD by regulating the PI3K-Akt pathway [28]. Additionally, many reports also demonstrate that the calcium signaling pathway acts as a pivotal regulator in PD development [29, 30]. The above results indicated that the identified DEMs might play crucial roles in PD by mediating ECM organization, dopamine biosynthesis, PI3K-Akt, and calcium signaling pathways.

It is well-established that circRNAs can bind to miRNAs via miRNA response element, and consequently upregulate expression of miRNA target genes, forming a ceRNA regulatory network [31]. circRNAs play a critical role in many neurological diseases and can serve as diagnostic biomarkers for these diseases, including PD [32]. Xiao et al. identified that a circRNA panel containing four differentially expressed circRNAs has a high diagnostic ability to distinguish PD patients from healthy controls [33]. Dysregulation of miRNAs has also been observed in the plasma and brain tissues of PD patients [34]. miRNAs can bind to the 3’UTRs of mRNAs via base-pairing, leading to either mRNA degradation or translation repression, thereby regulating various biological processes in PD [35]. Many studies have reported that the aberrant function of circRNA-miRNA-mRNA ceRNA networks regulates the onset and progression of PD. For example, Zhang et al. proposed that circ_0004381 targets the miR-185-5p/RAC1 axis to facilitate neuronal injury in PD [36]. Wang et al. presented that circSAMD4A is upregulated in midbrain tissues of PD mice and its knockdown attenuates dopaminergic neuronal apoptosis and autophagy by targeting miR‑29c‑3p [37]. circTLK1 aggravates dopaminergic neuron injury in PD by upregulating DAPK1 via competitively interaction with miR-26a-5p [38]. Herein, to investigate key genes involved in PD progression, we identified 21 hub genes via PPI network construction and established ceRNA networks with these hub genes. As a result, we found that 31 circRNAs could sponge miR-371a-3p to upregulate the hub gene *KCNJ6*. Additionally, although 20 other hub genes were not included in the ceRNA networks, some of them have also been reported to be dysregulated in PD, including *ESR1* [33], *DRD2* [39], *SLC18A2* [40], and *PITX3* [41]. These reports partially increase the credibility of our research.

*KCNJ6* (potassium inwardly rectifying channel subfamily J member 6), also known as *GIRK2*, belongs to G-protein-gated inwardly rectifying potassium channel family, which mediates various physiological processes via G-protein coupled receptor stimulation [42]. Evidence suggests that activation of *KCNJ6*/*GIRK2* attenuates Aβ-induced hyperactivity and subsequent neuronal death, thereby playing a neuroprotective role in Alzheimer’s disease [43]. Previous reports have demonstrated that *KCNJ6* is a dopaminergic neuron phenotype marker and exhibits a decreased expression level in substantia nigra of PD patients [24, 44], which is consistent with the microarray results in this study. Nonetheless, the molecular mechanism of *KCNJ6* in PD has not been clarified. Herein, we found that *KCNJ6* could be targeted by miR-371a-3p, which was shown to be markedly upregulated in PD samples. Previous reports have indicated the oncogenic role of miR-371a-3p in several cancers, such as colorectal cancer and germ cell cancer [45, 46]. However, studies on the role of miR-371a-3p in PD are lacking. In addition, our results showed that the circRNAs binding to miR-371a-3p were markedly downregulated in PD samples, indicating the potential role of circRNA/miR-371a-3p/KCNJ6 axis in the pathogenesis of PD. To our knowledge, our study is the first to show that these circRNAs are differentially expressed in PD compared to normal controls. Nevertheless, the specific functional roles of these circRNAs in PD remain unclear, highlighting the need for future investigations.

It is worth noting that there are some limitations in the present study. Firstly, differential expression analyses of circRNAs, miRNAs and mRNAs were conducted based on a single dataset with relatively small sample size, which may lead to selection bias. Secondly, our results are based only on bioinformatics predictions. Further experimental verification is required to determine the biological significance of the differentially expressed RNAs.

In conclusion, our study established a novel PD-related ceRNA regulatory network of circRNA-miRNA-mRNA via bioinformatics analysis. It was revealed that 31 circRNAs could competitively interact with miR-371a-3p to upregulate *KCNJ6* expression, thereby playing a potential role in PD progression. Our results may help improve the understanding of circRNA-mediated ceRNA regulatory mechanisms in PD pathogenesis and provide new molecular markers for early detection of the disease.

### Supplementary Information

**Supplementary Material 1.**

## Data Availability

The data and materials used to support the findings of this study are available upon request from the corresponding author.
