# Supplementary material for: Integrated bioinformatics analysis for exploring potential biomarkers related to Parkinson’s disease progression
Source: BMC Med Genomics. 2024 May 17;17:133. doi: 10.1186/s12920-024-01885-9 (PMC11100188; doi:10.1186/s12920-024-01885-9)
Supplement: Supplementary file 1 — Supplementary Material 1. [file 12920_2024_1885_MOESM1_ESM.docx]

**Table S1. Top ten downregulated circRNAs.**

| circRNA Name | Log2FC | Average Expression | t | p value | Regulation |
| --- | --- | --- | --- | --- | --- |
| hsa_circ_0138219 | -8.45205 | 9.555974 | -3.37327 | 0.003931 | Down |
| hsa_circ_0065842 | -7.15768 | 8.529635 | -3.37804 | 0.003892 | Down |
| hsa_circ_0115568 | -6.86993 | 7.98435 | -3.63516 | 0.002268 | Down |
| hsa_circ_0093281 | -6.84231 | 8.118871 | -3.4601 | 0.003276 | Down |
| hsa_circ_0067965 | -6.77164 | 7.91923 | -3.5651 | 0.002628 | Down |
| hsa_circ_0034762 | -6.70014 | 7.802477 | -3.59258 | 0.002481 | Down |
| hsa_circ_0085965 | -6.67147 | 7.825123 | -3.5857 | 0.002517 | Down |
| hsa_circ_0131191 | -6.5895 | 7.734765 | -3.61461 | 0.002369 | Down |
| hsa_circ_0037165 | -6.39527 | 7.715635 | -3.51714 | 0.002907 | Down |
| hsa_circ_0041511 | -6.33542 | 7.571096 | -3.55558 | 0.002681 | Down |

**Table S2. Top ten upregulated and downregulated miRNAs.**

| miRNA Name | Log2FC | Average Expression | t | p value | Regulation |
| --- | --- | --- | --- | --- | --- |
| hsa-miR-183-5p | 0.87 | 4.265833 | 3.478076 | 0.001594 | Up |
| hsa-miR-3195 | 0.800833 | 4.992083 | 2.476745 | 0.019247 | Up |
| hsa-miR-1260a | 0.539167 | 7.095417 | 2.129057 | 0.041747 | Up |
| hsa-miR-500b | 0.478333 | 3.716667 | 2.71002 | 0.011112 | Up |
| hsa-miR-204-5p | 0.425833 | 9.657917 | 2.835714 | 0.008193 | Up |
| hsa-miR-1245a | 0.416667 | 3.455 | 2.347859 | 0.025814 | Up |
| hsa-miR-219-1-3p | 0.411667 | 3.8275 | 2.647333 | 0.012907 | Up |
| hsa-miR-650 | 0.398333 | 3.414167 | 2.668202 | 0.012282 | Up |
| hsa-miR-122-5p | 0.39 | 3.741667 | 2.822444 | 0.008463 | Up |
| hsa-miR-554 | 0.384167 | 3.410417 | 2.388318 | 0.023561 | Up |
| hsa-miR-155-5p | -0.67083 | 4.88375 | -2.90611 | 0.00689 | Down |
| hsa-miR-219-2-3p | -0.54917 | 11.42542 | -2.46822 | 0.019629 | Down |
| hsa-miR-208a | -0.50083 | 4.01125 | -2.69907 | 0.011408 | Down |
| hsa-miR-588 | -0.42833 | 3.574167 | -2.63443 | 0.013309 | Down |
| hsa-miR-1224-3p | -0.4175 | 3.022917 | -2.55667 | 0.015985 | Down |
| hsa-miR-296-3p | -0.41683 | 2.79575 | -2.17303 | 0.037973 | Down |
| hsa-miR-920 | -0.38333 | 3.535 | -2.64096 | 0.013104 | Down |
| hsa-miR-875-5p | -0.37917 | 3.607917 | -2.82024 | 0.008508 | Down |
| hsa-miR-200b-3p | -0.365 | 4.216667 | -2.23173 | 0.033413 | Down |
| hsa-miR-3200-3p | -0.36 | 6.498333 | -3.42255 | 0.001845 | Down |

**Table S3. Top ten upregulated and downregulated mRNAs.**

| Gene Name | Log2FC | Average Expression | t | p value | Regulation |
| --- | --- | --- | --- | --- | --- |
| PKD1P5 | 4.234211 | 5.146151 | 2.256354 | 0.040026 | Up |
| HSFX2 | 3.984148 | 2.238896 | 4.163397 | 0.000897 | Up |
| IGSF3 | 2.704454 | 2.656664 | 2.20772 | 0.043895 | Up |
| CLDN19 | 2.516197 | 4.380212 | 2.173036 | 0.046866 | Up |
| MYLK4 | 2.368727 | 2.071204 | 2.349184 | 0.033512 | Up |
| MPZ | 2.349593 | 8.009877 | 2.316975 | 0.035649 | Up |
| PRX | 2.304657 | 6.3778 | 3.213323 | 0.006042 | Up |
| DCLK3 | 2.293527 | 4.292629 | 3.182906 | 0.006424 | Up |
| KIF24 | 2.155336 | 4.34561 | 2.320078 | 0.035438 | Up |
| PROX1 | 2.0625 | 4.548142 | 2.443846 | 0.027909 | Up |
| HSFX1 | -3.64009 | 4.082285 | -3.33027 | 0.004773 | Down |
| SLC6A3 | -2.77575 | 7.397669 | -3.46906 | 0.003607 | Down |
| SLC18A2 | -2.60171 | 8.488656 | -2.42011 | 0.029224 | Down |
| JUND | -2.54981 | 5.008884 | -2.17515 | 0.046679 | Down |
| OR7E125P | -2.45519 | 2.504249 | -2.34572 | 0.033735 | Down |
| ALDH1A1 | -2.10241 | 9.279369 | -6.70769 | 8.50E-06 | Down |
| PITX3 | -2.07757 | 3.211064 | -5.22137 | 0.000117 | Down |
| NTSR1 | -2.07553 | 5.904747 | -3.34911 | 0.004595 | Down |
| SLC35D3 | -2.02527 | 4.217661 | -2.80251 | 0.013775 | Down |
| SDC1 | -1.91769 | 4.42224 | -3.09361 | 0.00769 | Down |
